# *Rickettsia felis* in Fleas, Western Australia

**DOI:** 10.3201/eid1205.051458

**Published:** 2006-05

**Authors:** Drew Schloderer, Helen Owen, Phillip Clark, John Stenos, Stanley G. Fenwick

**Affiliations:** * Murdoch University, Murdoch, Western Australia, Australia;; † The Australian Rickettsial Reference Laboratory, Geelong, Victoria, Australia

**Keywords:** Australia, flea, Ctenocephalides, Rickettsia felis, dispatch

## Abstract

This study is the first confirmation of *Rickettsia felis* in Australia. The organism was identified from 4 species of fleas obtained from dogs and cats in Western Australia, by using polymerase chain reaction amplification and DNA sequencing of the citrate synthase and outer membrane protein A genes.

Several rickettsial diseases have been documented in Australia, namely, Queensland tick typhus (*Rickettsia australis*), scrub typhus (*Orientia tsutsugamushi*), murine typhus (*R. typhi*), and more recently, Flinders Island spotted fever (*R. honei*), as well as the closely related Q fever (*Coxiella burnetii*) and cat scratch fever (*Bartonella henselae*) (*1**,**2*). Cases of murine typhus have been reported in Western Australia (WA) since 1927, and a serologic survey provided evidence that members of the closely related spotted fever group (SFG) rickettsiae are also present in the state (*3**,**4*).

*R. felis* is a newly discovered species within the SFG; it is transmitted by fleas, which makes it unique within the biogroup. The species was first detected in the cat flea, *Ctenocephalides felis*, and subsequently has been determined to cause human disease in a number of countries (*5**–**8*). A recent study in New Zealand provided the first report of the organism in Oceania (*9*). Infected domestic and wild animals may not exhibit clinical disease and act as reservoirs of infection for humans. No definitive reports of the organism have been made in Australia, however, a study of cat fleas that used polymerase chain reaction and restriction fragment length polymorphisms of the amplification products (PCR-RFLP), provided strong evidence that *R. felis* exists (*10*). Our study aimed to confirm the presence of *R. felis* in Australia and to determine the distribution of the organism in WA. This study was approved by the Murdoch University Animal Ethics Committee.

## The Study

Samples were collected from 8 regional centers throughout WA: Esperance, Albany, Augusta, Manjimup, Busselton, Bunbury, Pinjarra, and Geraldton. Veterinarians from each site collected fleas from dogs and cats, preserved them in 70% ethanol, and sent them to Murdoch University for identification and analysis. The fleas were identified by using light microscopy. For each of the sampled 116 dogs and 43 cats, 1–5 fleas were pooled to increase the likelihood of finding rickettsial DNA. DNA was extracted from each flea pool by using a Qiagen QIAmp DNA minikit (Qiagen, Hilden, Germany) according to the manufacturer's instructions.

Initial PCR targeted the citrate synthase (*gltA*) gene, which is conserved across the genus *Rickettsia*. Primers and PCR conditions were sourced from the literature (*11*). Samples positive by the initial screening PCR were then selected for a second round of PCR targeting the outer membrane protein A gene (*ompA*), which is specific for spotted fever group (SFG) rickettsiae (*12*), and thus distinguishes *R. typhi* and *B. henselae*, which are also found in fleas and would be detected with the *gltA* PCR, from *R. felis*. PCR conditions were validated and optimized by using *R. typhi*- and *R. felis*-positive controls.

All PCR products were separated on a 1% agarose gel at 86 V for 30 min and visualized under UV light. Eight *C. felis* samples that were positive for both *gltA* and *ompA* were sequenced by using the *gltA* primers. Two of these samples were also sequenced by using *ompA* primers. The products of the PCR were extracted from the agarose for sequencing by using the Qiagen gel extraction kit (Qiagen) according to the manufacturer's recommendations. Purified PCR products were sequenced by using the Big Dye version 3.1 terminator kit (Applied Biosystems, Foster City, CA, USA) and the Applied Biosystems 373 automatic sequencer and were compared to those of previously characterized rickettsiae in GenBank by using BLAST (available from http://www.ncbi.nlm.nih.gov) analysis.

## Conclusions

A total of 368 fleas collected from 43 cats and 116 dogs were pooled into 165 flea pools (mixed infections from 6 animals meant 6 more flea pools than the total number of animals). Four different species of flea were identified: *C. felis* (49 from 38 cats and 241 from 99 dogs), *C. canis* (12 from 7 dogs), *Echidnophaga gallinacea* (4 from 3 cats and 57 from 16 dogs), and *Spilopsyllus cuniculi* (5 from 2 cats). Six dogs had a mixed population of fleas; 4 of these had *C. felis* and *E. gallinacea*, and 2 had *C. felis* and *C. canis*.

Forty-two (36%) of the 116 flea pools from dogs were positive for both the *gltA* and *ompA* genes. Similarly, 14 (33%) of 43 flea pools from cats were positive for both genes. Notably, positive samples were obtained from all the locations in the study, indicating widespread distribution throughout the state (Table).

**Table:** Flea species positive for rickettsiae from dogs and cats in Western Australia*

Location	Animal	No. animals	No. flea pools	Flea species	No. *gltA*+*ompA*+ (%)	Genes sequenced†
Broome	Dog	4	4	*Echidnophaga gallinacea*	2 (50)	ns
Geraldton	Cat	17	14	*Ctenocephalides felis*	6 (43)	ns
			3	*E. gallinacea*	1 (33)	ns
	Dog‡	38	30	*C. felis*	14 (46)	ns
			10	*E. gallinacea*	2 (20)	ns
Pinjarra	Cat	1	1	*C. felis*	0 (0)	
	Dog	7	7	*C. felis*	3 (43)	*gltA*
Manjimup	Cat	4	4	*C. felis*	1 (25)	ns
	Dog	6	6	*C. felis*	3 (50)	*gltA*
Bunbury	Cat	5	5	*C. felis*	2 (40)	ns
	Dog	6	6	*C. felis*	3 (50)	*gltA*
Busselton	Cat	10	8	*C. felis*	3 (38)	ns
			2	*Spilopsyllus cuniculi*	0	
	Dog§	33	28	*C. felis*	6 (21)	gltA, ompA
			7	*C. canis*	0	
			1	*E. gallinacea*	0	
Augusta	Cat	1	1	*C. felis*	0	
	Dog	2	2	*C. felis*	1 (50)	*gltA*
Albany	Dog	10	10	*C. felis*	5 (50)	*gltA*
Esperance	Cat	5	5	*C. felis*	3 (60)	ns
	Dog¶	10	10	*C. felis*	3 (30)	ns
			1	*E. gallinacea*	0	

*Fleas were collected from 116 dogs and 43 cats. 6 animals were co-infected with 2 different species of fleas as indicated. †ns, no sequencing performed on this sample; *gltA*–*gltA* segment sequenced; *ompA*–*ompA* gene segment sequenced. ‡Two dogs had *C. felis* and *E. gallinacea*. §One dog had *C. felis* and *E. gallinacea*, 2 dogs had *C. felis* and *C. canis*. ¶One dog had *C. felis* and *E. galli*

Of the 8 samples from *C. felis* positive for both *gltA* and *ompA* genes that were sequenced by using the *gltA* primers, all sequences matched the *gltA* gene from *R. felis* (99% similarity). Of the 2 samples that were also sequenced by using *ompA* primers, the sequences matched the *R. felis ompA* gene (100% similarity).

Our study demonstrates that *R. felis* is present in multiple sites in WA and was conclusively present in 1 of the 4 flea species collected (*C. felis*). The results obtained from 2 rounds of PCR are highly indicative of *R. felis* infection in *E. gallinacea* also. Because *C. felis* has the highest rate of infection and is prevalent, highly mobile, and nonspecific in its choice of hosts (including humans), it is likely to be the most important vector of the organism. *C. canis* has been identified as a vector of *R. felis* (*13*); however, this finding was not supported by our study. The presence of *R. felis* in *E. gallinacea* has been previously reported (*14*). To our knowledge, this is the first time a rickettsia has been detected from *S. cuniculi*, which could also be a potential vector for *R. felis*.

The significance of *R. felis* as a cause of human disease in WA has not yet been determined. Because of the often transient and nonspecific symptoms of rickettsioses, infections may not be readily detected. A serologic survey conducted in the Kimberley region of WA (*10*) showed evidence of scrub typhus and an SFG rickettsia, but no further work has identified the specific organism responsible for the latter. Another serologic study of 866 people throughout southwest WA showed evidence of infection with *R. typhi* (0%–1%) and another undetermined SFG rickettsia (3%–13%). During the same study, fleas were collected from cats and dogs in Perth and screened for rickettsiae by using PCR-RFLP of the *gltA* gene; the results provided evidence for the existence of *R. felis*. However, no sequencing data confirmed its presence (*4*).

The results from the current study showed that the *gltA* gene from all the sequenced samples most closely matched the *gltA* gene in the species *R. felis*. The identity of the sequenced samples as *R. felis* was confirmed by the *ompA* gene sequences. Therefore, the other samples shown to be positive for SFG rickettsiae in the PCR screening process are probably also *R. felis*.

This study has confirmed the presence of *R. felis* in WA; consequently, this rickettsial disease should be included as a differential diagnosis for influenzalike illnesses in persons who own or work with companion animals.
